# Sex Differences in Substance Use Disorders: A Neurobiological Perspective

**DOI:** 10.3389/fgwh.2021.778514

**Published:** 2021-12-08

**Authors:** Jennifer L. Cornish, Asheeta A. Prasad

**Affiliations:** ^1^School of Psychological Sciences, Faculty of Medicine, Health and Human Sciences, Macquarie University, Sydney, NSW, Australia; ^2^School of Psychology, University of New South Wales, Sydney, NSW, Australia; ^3^Faculty of Medicine and Health, School of Medical Sciences, University of Sydney, Sydney, NSW, Australia

**Keywords:** substance use disorder, methamphetamine, alcohol, nicotine, cocaine, cannabis, opiate, sex difference

## Abstract

Clinical studies provide fundamental knowledge of substance use behaviors (substance of abuse, patterns of use, relapse rates). The combination of neuroimaging approaches reveal correlation between substance use disorder (SUD) and changes in neural structure, function, and neurotransmission. Here, we review these advances, placing special emphasis on sex specific findings from structural neuroimaging studies of those dependent on alcohol, nicotine, cannabis, psychostimulants, or opioids. Recent clinical studies in SUD analyzing sex differences reveal neurobiological changes that are differentially impacted in common reward processing regions such as the striatum, hippocampus, amygdala, insula, and corpus collosum. We reflect on the contribution of sex hormones, period of drug use and abstinence, and the potential impact of these factors on the interpretation of the reported findings. With the overall recognition that SUD impacts the brains of females and males differentially, it is of fundamental importance that future research is designed with sex as a variable of study in this field. Improved understanding of neurobiological changes in males and females in SUD will advance knowledge underlying sex-specific susceptibility and the neurobiological impact in these disorders. Together these findings will inform future treatments that are tailor designed for improved efficacy in females and males with SUD.

## Introduction

Disorders of substance use such as alcohol, psychostimulants, opioids, and cannabis are prevalent in both males and females (1). However, over the last decade, the rate of increase in substance use disorders (SUD) was significantly greater in females when compared to males (2). For example, cases of alcohol use disorder (AUD) in females increased by 84% compared to 35% in males (3), and it has been reported that females escalate their psychostimulant use faster than males (4). These data clearly demonstrate a greater need for improved understanding of sex differences in SUD.

Clinical studies in SUD provide fundamental knowledge in substance use behaviors (substance of abuse, patterns of use, relapse rates) and associated changes to neural structure and function. Historically, female participation in scientific studies is less than males (5). However, while the enrolment of females in clinical SUD studies has increased over the years, the number of females represented in these studies is still lower than males. In part, this lack of female participation reflects a “one treatment for all” approach and the logistical limitations of child rearing and child care responsibilities (6). Within the last decade, females have been included in more than 50% of neural imaging studies (7), and despite the recognition that sex differences are likely to exist in disease etiology, pathology, and successful treatment, not all studies incorporate an analysis of sex as a variable, nor have sex-stratified analyses been conducted.

For this review we have aimed to explore sex differences that exist at a structural level in the brain following dependence on common drug types. It is important to note that we define sex as male and female and not the social construct of gender. Previous research investigating sex differences in the cortex across a group of combined users (stimulants, nicotine, alcohol, heroin, cannabis) has highlighted that the insula cortex is thinner in females than males when compared to healthy sex-matched controls (1). This prompted the question of the relationship between the primary drug used and brain structural changes between male and female SUD, and if they are common or distinct. Common approaches to examine changes in brain structure and function in SUD are neural imaging techniques of magnetic resonance imaging (MRI), functional magnetic resonance imaging (fMRI), and positron emission tomography (PET). Here, we describe and discuss the neurobiological differences between males and females in specific SUD, in order to understand the key changes that are unique to females to aid the development of therapies for successful treatment.

## Neural Circuitry of Substance Use Disorders

In SUD research, much of what we have studied in relation to brain circuitry that drives continued drug use has been through rodent or primate models. More often than not, these findings have translated to human neurobiology (8). In a general sense, the neurobiology of SUD has focused on function of the dopamine reward and motivation pathways, extending from the ventral midbrain to striatal areas, with recruitment of cortical areas as the disorder progresses, highlighting dysfunctional executive control. The hippocampus, amygdala, bed nucleus of the stria terminalis (extended amygdala), and insula are also key components of maintaining substance use, altering feedback to motivational circuits from memory processing, stress reactivity, and interoceptive states, respectively. A recent mega analysis of gray matter structural changes in SUD noted thinning of brain regions, with two structures of the insula and medial orbitofrontal cortex (OFC) common to all SUDs (9). The key focus of the current review, however, is to identify sex differences in neural structures that are associated with specific SUDs. While it is recognized that some sex differences exist between brain sizes in healthy individuals, we have only included those studies that compare brain changes following SUD of each sex to those of healthy same-sex controls, unless stated otherwise. Henceforth, comparisons of sex are listed as relative changes between SUD and healthy same-sex controls for each sex, and not in absolute terms.

## Alcohol

Alcohol is the most widely used substance of abuse (10). Epidemiological studies show the rate of AUD has significantly increased in females more than males over the last 10 years (2). Among substances of abuse, alcohol is most prominently used in females (3, 11). In general, alcohol dependence has been associated with changes in brain volume in reward circuitry including the cortex, insula, and hippocampus (9, 12).

There are some MRI studies that do not detect sex dependent changes in brain regions in AUD (13–15), however others show clear sex differences in regions including the hippocampus, cerebellum, and corpus callosum. Analysis of sex interactions revealed opposite effects in males and females, where the volume of these reward areas in AUD males were smaller than non-alcoholic males, whereas the reward regions were larger in AUD females compared to non-alcoholic females (16). This bi-directional effect in sexes was also measured as larger volume in the corpus callosum and the superior longitudinal fasciculi in females with AUD which was decreased in male AUD compared to sex-matched controls (17, 18). Other studies have identified sex dependent changes in amygdala and hippocampal volume. Sex-interactions showed alcohol-dependent males had 6% smaller right amygdala volume than control males, while this effect was not clearly detected among females. Males with alcohol dependence had smaller volumes of the total amygdala and its basolateral nucleus than male controls, that exacerbated with alcohol dose (19). Hippocampus size was also reduced more in males than female AUD cases (20). In general, it appears that male AUD cases frequently report reduced volume in reward regions, that is either opposite or not detected in female AUD when compared to sex matched controls, with females often reporting larger volume than sex-matched controls.

## Nicotine

It is traditionally reported that males engage with smoking more than females (21). With the cessation of cigarette smoking, electronic cigarette use, and vaping of nicotine is on the rise (22). Recent research by Lin et al. (23) described a thorough study to identify sex specific differences in cigarette smoking. This study included non-smoker and smoker groups, of which 40% were female participants. Structural and resting-state functional MRI data identified smaller volume of the right amygdala in female smokers when directly compared to male smokers. The amygdala volume was negatively correlated with impulsivity scores in female but not male smokers, with previous studies demonstrating that female smokers were more impulsive compared to males (24). Such studies demonstrate modification of amygdala volume and impulsive traits specifically in females. Interestingly, the volume of the left caudate was specifically reduced in the male smokers but not in female smokers. Despite this reported decrease in size, it was determined that an increase in the resting state functional connectivity existed between the left caudate and the left prefrontal cortex specifically in male smokers (23). This may be the result of enhanced dopamine release produced by smoking in males, and not females, as measured by PET (25).

## Cannabis

Legalization of cannabis use for medicinal and recreational purposes is increasing globally, impacting on the rates of cannabis use disorder (CbUD) (26). The prevalence of cannabis use and CbUD is higher in males than females (27), however during periods of abstinence, females are reported to experience stronger cannabis withdrawal symptoms when compared to males (28). A MRI study in CbUD reported that, similar to AUD, amygdala volume was smaller in individuals who use cannabis, yet was not mediated by sex (29). Among other brain volumes examined, smaller cerebellar volume was reported in those who use cannabis compared to controls and this effect was greater in females (30). Another recent study examined the cannabis consumption behavior and fMRI in CbUD subjects; participants were sex-matched with sex ratio of 13:12 (male: female) yet analysis of sex difference was not reported (31).

## Methamphetamine

General neuroimaging results following methamphetamine use disorder (MUD) have recently been reviewed (32) and extended (33–36), consistently reporting alterations to corticostriatal regions. Notably there are structural and/or functional changes to insula, inferior and precentral gyrus, striatum, and frontal cortex. When investigating the neurobiological impact of MUD on different sexes, studies have indicated sex-specific changes to the striatum, hippocampus, and cortical areas (37).

The acute exposure to amphetamine produces a greater striatal level of dopamine in males when compared to females (38). Using ultrasound imaging of the midbrain of subjects with a history of methamphetamine use (5+ occasions), the dopamine rich region of the substantia nigra was enlarged, which was greater in males than females, when compared to healthy controls (39). In contrast, MRI comparisons in individuals who currently use methamphetamine, detected that the right ventral striatum (nucleus accumbens) was significantly larger in female users when compared to controls, which was not evident in male users (37). Structural MRI of recently abstinent MUD measured enlarged striatal regions (putamen and globus pallidus) in both sexes, with females also showing an increase in the mid-posterior corpus callosum, when compared to controls (40). These data highlight changes to dopamine rich regions by amphetamine type stimulants, that may be differentially impacted in males and females.

The study by Kogachi et al. (37) also demonstrated cortical differences between sexes in MUD, and correlated this with impulsive control. Here, it was shown that the volume of the right superior frontal cortex was much smaller in female, and larger in male MUD, when compared to sex-matched controls. Moreover, these sex-dependent volume changes to this area in MUD were associated with greater levels of impulsivity.

Abstinent MUD have also shown a sex dependent difference in hippocampal size (right) when compared to controls (41). The hippocampus from control individuals was greater in females when compared to males, however in abstinent MUD, males and females had similar right hippocampal volume. This equivalent absolute sizing was the result of methamphetamine induced reduction in the female hippocampal volume when compared to healthy controls. It should be noted, however, that the females were more recently abstinent (average of 112 days) than the males (average 192 days), providing less time for any recovery of hippocampal size.

## Cocaine

The neurobiological differences between males and females in cocaine use disorder (CUD) has been reviewed by Anderson et al. (42). From this review it was determined that females may be more resistant to neural damage induced by cocaine use, and in some instances showed opposite activity to males in brain regions such as the medial OFC and superior frontal gyri (reduced), or anteriolateral temporal or anterior cingulate cortex (ACC) (increased) (43). In addition, using single-photon emission computed tomography (SPECT), female cocaine-dependent subjects had increased activity in the posterior cingulate gyrus, whereas males had reductions in activity in the precentral, superior, frontal, and anterior cingulate gyrus (42, 44). Following from the review by Anderson et al., reduced gray matter was detected in the insula, superior temporal gyrus, left inferior frontal gyrus, and hippocampus in female CUD, while male CUD subjects had reduced gray matter volume in the precentral gyrus and mid cingulate cortex (45). On a functional level, it has further been shown that error processing in the dorsal ACC similarly predicted relapse to cocaine use in both sexes, however activation of the thalamus predicted relapse in females, while the insula predicted relapse in males (46).

## Opiates

Neuroimaging findings for opiate use disorder (OUD) has recently been reviewed by Moningka et al. (47). Briefly they report that although the frequency of OUD is different between males and females, very few studies (<15%) used both sexes in their sample, with none investigating sex differences in OUD neurobiology. Exposure to opiate cues in opiate users indicates activity in expected brain regions across reward and motivation circuitry; in midbrain, limbic, prefrontal, orbitofrontal, and parietal cortices.

## Perspectives

Despite limited research with inclusion of females and analysis of sex interactions, application of neural imaging studies report sufficient evidence of neurobiological differences between males and females in SUD (1, 7). Overall, the sex differences in brain regions most impacted in SUD suggest distinct alterations in reward and motivation processing, where selective changes were reported in striatum, hippocampus, amygdala, insula, and corpus collosum, depending on primary substance of use (16).

The striatum is a central region in reward processing and alteration in striatal size was common to smokers and those with MUD. Male smokers demonstrated a reduction in caudate size, and female MUD measured an increase in ventral striatal size when compared to sex-matched controls. It was also reported that enhanced dopamine release occurred in male smokers or following amphetamine use when compared to females, indicating sex dependent regulation of reward neurotransmission.

As a critical region that influences memory on motivational circuits in driving continued drug use, the hippocampus was opposingly affected in AUD when compared to MUD or CUD. Relative to healthy controls, hippocampus size was significantly reduced in females when compared to males following psychostimulant use, however the hippocampus of males appeared to be significantly more reduced in size when compared to females in AUD.

In AUD the amygdala was consistently reduced in males when compared to females, however the opposite was measured for nicotine, with reduced right amygdala size measured in females when compared to healthy controls. These findings suggest that emotional and stress reactivities encouraging drug use may be differentially expressed between the sexes in AUD and smokers.

The insula provides interoceptive feedback to motivational circuits and was identified as thinner in female SUD in general, with the current review reporting that reduced gray matter was also measured in female CUD when compared to males, relative to controls. In addition to gray matter abnormalities, alterations to the white matter tract of the corpus collosum (48) was reportedly different between the sexes in AUD and MUD, both deemed larger in females than males.

In addition to common brain regions impacted by SUD, recent findings reported changes in periaqueductal gray (PAG) connectivity in CUD. Cocaine craving was positively correlated with PAG-ventromedial prefrontal cortex (vmPFC) connectivity in males, yet the reverse was true for females (49), however noting that healthy controls were not compared to in this study. There are other brain regions involved in SUD such as the subthalamic nucleus (50) and ventral subiculum (51, 52), that are yet to be investigated with respect to sex dependent analysis. Such studies could shed light on additional brain regions critically important for female SUD therapies.

This review has focused on sex-dependent structural changes that result from SUD, and of course structural change does not necessarily mean functional change. There are some reports that have identified sex-dependent functional changes in SUD, particularly when craving has been elicited by drug or cue. For example, upon alcohol administration, while activity of the ACC was reduced in general in AUD cases (53, 54), sex differences analyzed in both studies produced conflicting results. Following the presentation of alcohol cues to alcohol drinkers, the ACC, amygdala, insula, thalamus, and the putamen were activated in both sexes, with activation of the left amygdala greater in males in comparison to females (55). The greater activity in the amygdala in males contrasts with the identification of reduced size of the amygdala in males when compared to females in AUD (19), also noting that there were a number of studies that did not support amygdala differences between the sexes in AUD. In CUD, males and females differ in the type of trigger to elicit activity in the amygdala, insula, and striatum, with their activity greater following drug cues in males compared to stress cues in females (56).

These studies highlight the importance of not only the structural differences between the sexes in SUD but also how the function of these regions change across the facets of maladaptive behavior. It is also recognized that structural change does not account for neurochemical or molecular change. For example, receptor level differences exist between the sexes in SUD, with the function of β2-nicotinic acetylcholine receptors altered between male and female abstinent smokers (57).

Identification of structural changes between the sexes with SUD is a small yet important step in our understanding of distinct neurobiological changes associated with drug use. These findings underpin the need for future studies to correlate structural change with both molecular alterations and executive and memory processes to fully understand the neurobiology driving substance use behaviors, using sex-stratified analyses.

There are a number of limitations that are apparent within this review that need consideration. The first is the role of female hormones in any analyses undertaken in SUD, as there is a clear lack of consideration of the effect of menstrual cycle on structural change in current SUD studies. One of the key differences between sexes was reduced dopamine release in the striatum of females compared to males following methamphetamine or nicotine administration (25, 38). Both clinical and pre-clinical studies report that dopamine release in the striatum is significantly reduced in female SUD cases in contrast to males. There is a strong correlation between increase levels of estradiol and greater alcohol consumption (58), where the impact of estradiol on alcohol consumption is thought to be mediated by dopamine levels (59). Preclinical studies have demonstrated links between estrous cycle and basal dopamine concentration, showing that dopamine levels are positively correlated with estradiol levels and negatively correlated with progesterone levels (60, 61). In addition, using a monetary reward paradigm and fMRI analysis, Dreher et al. (62) identified specific changes to the activity of reward circuitry that were mediated by different stages of the menstrual cycle (62). Clearly, the neurobiological impact of SUD in males and females is more complex than structural changes to brain regions, and the functional states of reward circuits may be dependent on sex hormones at the time of measure. The inclusion of analyses of sex hormones in future clinical/imaging studies are essential in determining neurobiological responses to SUD to better inform treatment between sexes.

The second limitation is the methodological differences that are inherent in comparing neuroimaging studies, including the imaging technique, the regions of interest studied, how they are analyzed and modest sample sizes. There is also the consideration of causality of SUD on neurobiology, demanding at least comparison to sex-matched healthy controls. Another consideration is the timing of when the neuroimaging has occurred and the length of abstinence period prior to measures in each sex. In line with this, a recent study has identified that the male brain appears more responsive to recovery following MUD abstinence than female brains (63). Using gray matter measures, they determined that abstinence length (average 120 days) increased volume of the orbitofrontal, parietal cortices, and hippocampus of male brains, which was not detected in females who had an average of 300 days abstinent. In a MRI study investigating gray matter volume in abstinent stimulant users (cocaine and/or amphetamines), volume changes were only detected in female users, and not males when compared to healthy controls (64). This may have been the result of a long abstinence period with less recovery in female users. The participants had been abstinent for more than a year, with reduction in gray matter volume in the regions spanning the frontal lobe, limbic regions, temporal lobe, and inferior parietal lobule only detected in dependent females. The lack of alteration in gray matter volume in male dependent participants aligns with faster recovery when compared to females, in line with Nie et al. (63). Future studies would benefit from longitudinal analyses of brain change over time following abstinence from substance use.

Last, but not least, is the recognition that most substance users are polysubstance users, making investigations of the effect of particular drugs on structural neurobiology in SUD fraught with variability. However, the overarching aim is to discover common themes of neurobiological impact of each SUD, to enable the development of targeted therapies depending on the primary substance used. Alternatively, increasing our knowledge of common structural and functional changes that occur in males or females across all SUD could allow for the identification of sex-dependent targets for individuals with high levels of polysubstance use.

The studies presented in this review have demonstrated the common brain areas of striatum, hippocampus, amygdala, insula, and corpus collosum that are differentially affected between males and females, depending on the primary SUD. These studies underscore a greater need for future research to include sex dependent analyses for tailored and effective treatment of SUDs.

## Author Contributions

All authors have contributed equally to the research, preparation, and editing of the manuscript.

## Funding

Preparation of this manuscript was supported by grants from the Australian Research Council (DE170100509) to AP.

## Conflict of Interest

The authors declare that the research was conducted in the absence of any commercial or financial relationships that could be construed as a potential conflict of interest.

## Publisher's Note

All claims expressed in this article are solely those of the authors and do not necessarily represent those of their affiliated organizations, or those of the publisher, the editors and the reviewers. Any product that may be evaluated in this article, or claim that may be made by its manufacturer, is not guaranteed or endorsed by the publisher.
